# A *Pseudomonas aeruginosa* EF-Hand Protein, EfhP (PA4107), Modulates Stress Responses and Virulence at High Calcium Concentration

**DOI:** 10.1371/journal.pone.0098985

**Published:** 2014-06-11

**Authors:** Svetlana A. Sarkisova, Shalaka R. Lotlikar, Manita Guragain, Ryan Kubat, John Cloud, Michael J. Franklin, Marianna A. Patrauchan

**Affiliations:** 1 Department of Microbiology and Molecular Genetics, Oklahoma State University, Stillwater, Oklahoma, United States of America; 2 Department of Microbiology, Montana State University, Bozeman, Montana, United States of America; 3 Center for Biofilm Engineering, Montana State University, Bozeman, Montana, United States of America; Universitätsklinikum Hamburg-Eppendorf, Germany

## Abstract

*Pseudomonas aeruginosa* is a facultative human pathogen, and a major cause of nosocomial infections and severe chronic infections in endocarditis and in cystic fibrosis (CF) patients. Calcium (Ca^2+^) accumulates in pulmonary fluids of CF patients, and plays a role in the hyperinflamatory response to bacterial infection. Earlier we showed that *P. aeruginosa* responds to increased Ca^2+^ levels, primarily through the increased production of secreted virulence factors. Here we describe the role of putative Ca^2+^-binding protein, with an EF-hand domain, PA4107 (EfhP), in this response. Deletion mutations of *efhP* were generated in *P. aeruginosa* strain PAO1 and CF pulmonary isolate, strain FRD1. The lack of EfhP abolished the ability of *P. aeruginosa* PAO1 to maintain intracellular Ca^2+^ homeostasis. Quantitative high-resolution 2D-PAGE showed that the *efhP* deletion also affected the proteomes of both strains during growth with added Ca^2+^. The greatest proteome effects occurred when the pulmonary isolate was cultured in biofilms. Among the proteins that were significantly less abundant or absent in the mutant strains were proteins involved in iron acquisition, biosynthesis of pyocyanin, proteases, and stress response proteins. In support, the phenotypic responses of FRD1 Δ*efhP* showed that the mutant strain lost its ability to produce pyocyanin, developed less biofilm, and had decreased resistance to oxidative stress (H_2_O_2_) when cultured at high [Ca^2+^]. Furthermore, the mutant strain was unable to produce alginate when grown at high [Ca^2+^] and no iron. The effect of the Δ*efhP* mutations on virulence was determined in a lettuce model of infection. Growth of wild-type *P. aeruginosa* strains at high [Ca^2+^] causes an increased area of disease. In contrast, the lack of *efhP* prevented this Ca^2+^-induced increase in the diseased zone. The results indicate that EfhP is important for Ca^2+^ homeostasis and virulence of *P. aeruginosa* when it encounters host environments with high [Ca^2+^].

## Introduction

*Pseudomonas aeruginosa* is a facultative pathogen and a leading cause of severe nosocomial infections in both immunocompetent and immunocompromised patients [1] [2], including patients in intensive care units. *P. aeruginosa* is one of the primary organisms that forms biofilms on airway mucosal epithelium of patients with cystic fibrosis (CF) where it contributes to airway blockage and cellular damage. *P. aeruginosa* also causes infective endocarditis and device-related infections with high morbidity and mortality rates [3], [4], [5]. *P. aeruginosa* biofilm infections are increasingly difficult to treat with traditional antibiotic therapy, and are often not eradicated by host defensive processes [6], [7].

Calcium (Ca^2+^) is a well-known signaling molecule that regulates a number of essential processes in eukaryotes [8]. Slight abnormalities in cellular Ca^2+^ homeostasis have been implicated in many human diseases, including diseases associated with bacterial infections, such as CF pulmonary infections and endocarditis. Ca^2+^ metabolism is recognized to be central to the pathology of CF [9]. Ca^2+^ is a part of a hyperinflamatory host response to bacterial infection, and accumulates in airway epithelia, pulmonary and nasal liquids of CF patients [10], [11]. There is growing evidence suggesting that Ca^2+^ also plays a significant role in the physiology of certain bacteria, affecting maintenance of cell structure, motility, chemotaxis, cell division and differentiation, gene expression, transport, and spore formation [12], [13], [14], [15], [16]. Several bacteria including *P. aeruginosa* have been shown to maintain intracellular Ca^2+^ at sub-micromolar levels and produce Ca^2+^ transients in response to environmental and physiological factors [17]–[19]. However, molecular mechanisms of Ca^2+^ regulation in prokaryotes are not well defined.

In eukaryotes, the regulatory effects of Ca^2+^ are carried out by Ca^2+^-binding proteins (CaBPs), which may function as Ca^2+^ sensors, signal transducers, Ca^2+^ buffers or Ca^2+^-stabilized proteins. Prokaryotic genomes also encode CaBPs with different Ca^2+^-binding motifs, including the EF-hand motif [12], [20], which typically consists of a Ca^2+^-binding loop flanked by two α-helices. Acidic amino acids in the loop are preferentially bound by Ca^2+^ [21], and are responsible for Ca^2+^ - induced conformational changes required for function [22]. Bacterial EF-hand proteins constitute a majority of all studied CaBPs [23], and include the Ca^2+^ transducer calsymin CasA from *Rhizobium etli* [24], putative Ca^2+^ buffers and stabilizers calerythrin from *Saccharopolyspora erythraea* [25] and CabC [26] CabB [27] from *Streptomyces coelicolor*, and *Escherichia coli* transglycosylase MltB [28]. Most bacterial proteins containing Ca^2+^- binding motifs are classified as hypothetical proteins with unknown physiological functions.

Previously, we showed that Ca^2+^ modulates proteome profiles of *P. aeruginosa*, influences biofilm architecture, and alters production of several secreted virulence factors [29], [30]. Michiels et al [31] screened 31 bacterial genomes for CaBPs, and predicted that the *P. aeruginosa* hypothetical protein, PA4107, contains EF-hand Ca^2+^-binding motifs. We also identified PA4107 as having sequence similarity within the EF-hand motif to CasA of *R. etli*. Here, we further analyzed PA4107, which we designate EfhP (EF hand protein) and hypothesized that it plays roles in *P. aeruginosa* response to Ca^2+^. We generated deletion mutants lacking *efhP* in *P. aeruginosa* strain PAO1 and in the CF clinical isolate, FRD1, and studied their Ca^2+^-dependent phenotypes by global quantitative proteomics. Since certain stress response proteins had reduced abundances in the proteomes of the *efhP* deletion strains, we assayed the mutants for resistance to chemical and oxidative stresses. We also characterized the effect of the mutation on pyocyanin production and survival at low iron. Finally, we tested Ca^2+^- induced virulence of the mutant and wild-type strains in a plant infection model. The results indicate that EfhP is involved in plant virulence and in Ca^2+^- enhanced adaptation of *P. aeruginosa* to host environments.

## Materials and Methods

### Bacterial Strains, Media, and Growth Conditions

*Pseudomonas aeruginosa* FRD1 and PAO1 were used in this study. *P. aeruginosa* FRD1 is an alginate-overproducing (mucoid) CF pulmonary isolate [32], and *P. aeruginosa* PAO1 is the non-mucoid strain used for the original genome sequencing study [33]. Biofilm minimal medium (BMM) [30] contained (per liter): 9.0 mM sodium glutamate, 50 mM glycerol, 0.02 mM MgSO_4_, 0.15 mM NaH_2_PO_4_, 0.34 mM K_2_HPO_4_, and 145 mM NaCl, 20 µl trace metals, 1 ml vitamin solution. Trace metal solution (per liter of 0.83 M HCl): 5.0 g CuSO_4_.5H_2_O, 5.0 g ZnSO_4_.7H_2_O, 5.0 g FeSO_4_.7H_2_O, 2.0 g MnCl_2_.4H_2_O). Vitamins solution (per liter): 0.5 g thiamine, 1 mg biotin. The pH of the medium was adjusted to 7.0. The level of Ca^2+^ in BMM was below the detection level when measured by QuantiChrom™ calcium assay kit. CaCl_2_.2H_2_O when used was added to final concentration 5 or 10 mM. For experiments with no iron, trace metal solution contained no FeSO_4_.7H_2_O, and the corresponding BMM was referred to as no iron BMM (BMM-NI). The level of total Fe in BMM-NI was below the detection level when measured by Ferrozine assay [34].

For quantitative growth assays, cultures were pre-cultured in 5 ml tubes to mid-log phase, diluted to obtain optical density OD_600_ of 0.3, and used to inoculate 100 ml of BMM medium in 250-ml flasks. Growth data are based on at least 3 biological replicates. Biofilm formation was quantified using abiotic solid surface assay in 96-well plates as described previously [35]. Prior to inoculation, overnight cultures were diluted to obtain equal optical densities. Dilutions 1∶100 of the normalized cultures were inoculated into BMM medium and incubated for 24 h at 37 °C, the medium was replaced after 12 h. Nonattached cells were removed by washing the plates three times with saline solution (0.85% NaCl). Biofilms were stained with 1% crystal violet, non-bound crystal violet was removed by washing with water. Bound crystal violet was extracted with 80% ethanol/20% acetic acid, and the absorbance was measured at 595 nm using a microtiter plate reader (Tecan Instruments Inc.).

### Sequence Analyses and Gene Deletion

The putative EF-hand protein was first identified from the *Pseudomonas* genome database [36] by BLASTP searches using CasA of *Rhizobium etli* as the query [24]. Additional sequence and structural homology searches were performed using NCBI nr, RefSeq [37] UniProtKB/Swiss-Prot [38] and HHPred [39]. Functional domains were predicted using PFAM [40], and PROSITE [41]. The putative signal peptide of PA4107 was predicted using TMHMM 1.0 [42], SignalIP 4.0 [43], PrediSi [44] and SVMTM [45].

PA4107 (*efhP*) was deleted from the chromosomes of PAO1 and FRD1 and replaced with the gentamicin omega fragment using the allelic exchange strategy as described previously [30]. The primers used for allelic exchange were: EfhPEco47For (5′-CCCGGGAGCGCTCCTTGATCGGCGGGC-3′), EfhPBamH1Rev (5′-ACGGATCCGAGCAGGCTGGCGGAAGTCTTTTG-3′), EfhPBamH1For (5′-GCGGATCCAGCACTGAGCCTTTCCACAC-3′), and EfhPEco47Rev (5′-GCGTCGAGCGCTCCGGGCGCACTCCGCG-3′). The deletions mutants were complemented in trans by ligating the *efhP* PCR product into the XbaI restriction site, behind the P*_trc_* promoter of pMF36 [46]. Primers used to generate the *efhP* PCR product were: PA4107 Xba 5′ (5′-TCTAGACCGGCCGCGTTTACTGTGGAG-3′) and PA4107 Xba 3′(5′-TCTAGAGGGCGCCGCCAGGGTGTGG-3′). The resulting plasmid, pMF470, was introduced into the mutant strains by triparental mating using the pRK2013 conjugation helper plasmid.

### Two-dimensional Gel Electrophoresis of Cellular Proteins

Pharmalyte 3–7 and Immobiline Dry-Strips were purchased from GE Healthcare (Baie d’Urfé, Canada). Iodacetamide and 3-[(3-cholamidopropyl) dimethylamonio]-1-propanesulfonate (CHAPS) were from Acros Organics (New Jersey, NJ) and MP Biomedicals (Aurora, OH). All chemicals were of analytical grade.

Cells from planktonic and biofilm cultures were obtained as described previously [30]. Cell pellets were washed twice with saline solution and resuspended in TE buffer (10 mM Tris-HCl, 1 mM EDTA, pH 8.0, containing 0.3 mg/ml of phenylmethylsulfonyl fluoride (PMSF)). Cells were disrupted by sonication (12 times for 10 s, 4W, 4°C), and the cell debris and unbroken cells were removed by centrifugation (12000 g, 60 min, 4°C). The protein concentrations of the supernatants were determined by using the modified Lowry assay (Pierce, Thermo Scientific). Protein samples were stored as aliquots at –80°C. Two-dimensional gel electrophoresis was performed as described previously [30]. Briefly, proteins were loaded by in gel rehydration into 18 cm immobilized pH gradient (IPG) strips with pH range of 4–7. Solubilization buffer consisted of 9 M urea, 2 M thiourea, 4% CHAPS, 2% w/v carrier ampholytes, 0.037 M DTT, and a trace amount of bromphenol blue. Isoelectric focusing was conducted using a Multiphor II (Pharmacia) at 20°C. Proteins were focused for a total of 28 kVh. Second dimension electrophoresis was carried out on 11% polyacrylamide gels (230×200×1 mm) using vertical Hoefer Dalt System (Pharmacia) at 10°C using 10 mA/gel for 4 h, then 40 mA/gel for 14–16 h. Proteins were detected by Colloidal Coomassie staining.

### Proteomic Analysis

The gels were imaged, and the digital images were analyzed by using Progenesis Workstation Software (Nonlinear Dynamics, Durham, NC). A minimum of two biological replicates were performed for each condition, and the signal intensity of each spot was averaged over the replicates. The signal intensities (volumes) of protein spots were normalized against total signal intensity detected on a gel (normalized volumes). For protein identification we targeted proteins that met two criteria: (1) the protein had a normalized volume (NV) greater than 0.03, and (2) the proteins were at least threefold more or less abundant in the planktonic or biofilm proteomes cultivated with 10 mM Ca^2+^ versus their matched counterparts cultivated without added Ca^2+^. Proteins of interest were excised from the gels and identified based on peptide mass fingerprint analyzed on 4700 MALDI-TOF/TOF Mass spectrometer (University of Texas, Biomolecular Resource facility, Mass Spec Lab), using the Applied Biosystems GPS (version 3.6) software, MASCOT search engine and NCBInr database. A protein was considered identified if the hit fulfilled four criteria: (1) it was statistically significant (a MASCOT search score above 75), (2) the number of the matched peptides was at least five, (3) the protein sequence coverage was above 20%, and (4) the predicted molecular mass and pI were consistent with the experimentally determined values.

### Pyocyanin and Alginate Assays

For pyocyanin analysis, chloroform extraction of the pigment followed by a spectrophotometric assay was used as described in [47]. Briefly, BMM grown mid-log cultures of *P. aeruginosa* (100 µl) were grown on BMM agar for 24 h, and collected using 3–5 ml of saline. The samples were extracted with 3 ml of chloroform followed by extraction with 1 ml of 0.2 N HCl. The absorbance was measured at 520 nm, and pyocyanin concentrations (µg/ml) were calculated by multiplying the absorbance by extinction coefficient 17.072 [47]. The data were normalized by total cellular protein. For alginate assays, BMM-NI grown mid-log cultures of *P. aeruginosa* were grown on BMM-NI agar plates for 24 h. To deprive the cells of iron, seven passages onto fresh BMM-NI agar were performed. The cells were collected using 3–5 ml of saline. Alginate was precipitated with 2% cetyl pyridium chloride then isopropanol, dissolved in saline, and detected by using the modified carbazole method described in [46]. The concentration of alginate was determined using sodium alginate (Spectrum) as a standard and normalized by total cellular protein. The measurements were obtained for at least three independent replicates, and the averaged values with standard deviation are presented.

### Response to Chemical and Oxidative Stresses

The sensitivity of cells to stress was tested by exposing cultures to 1% ethanol, 10% DMSO, 1 mM H_2_O_2_ for 1 h or heat shock at 50°C for 30 min. The concentrations and the duration of treatments were optimized to reach approximately 50% cell survival in wild type FRD1. Prior to exposure, the cultures were grown to mid-log phase in BMM containing 10 mM CaCl_2_ or no added Ca^2+^, then diluted to obtain optical density of 0.2 at 600 nm. Viable cells were determined after serial dilution as colony form units (CFUs) before and after exposure. Results are expressed as the mean survival percentages from three independent experiments.

### Plant Virulence Assays

The lettuce infection model with the following modifications was used to assess strains pathogenicity [48]. Organic romaine lettuce was purchased fresh from market. Healthy leaves were detached, washed in 0.1% bleach and rinsed twice with distilled water and once with DI water. Midribs were cut and placed in Petri dish containing Whatman No.1 filter paper soaked in 10 mM MgSO_4_. *P. aeruginosa* wild type and mutant strains were incubated for 18 hours in BMM with 5 mM CaCl_2_ or with no added Ca^2+^. Cells were harvested, washed and resuspended in 10 mM MgSO_4,_ containing the same amount of Ca^2+^ as the original culture to obtain an OD_600_ of 0.2. One end of the midrib was inoculated with the resuspended culture and the other end of the midrib was inoculated with sterile 10 mM MgSO_4_ with 5 mM Ca^2+^ or no added Ca^2+^, as controls. The Petri dishes were placed in a clear plastic bin with water to maintain humidity. The bins were incubated at room temperature with ambient sunlight for six days after which the zones of disease were measured. The experiments were performed in at least three independent biological replicates, and the averaged values with standard deviation are presented.

### Estimation of Free Intracellular Calcium ([Ca^2+^]_in_)

PAO1 and its *efhP* lacking mutant PAO1043 were transformed with pMMB66EH (courtesy of Dr. Delfina Dominguez), carrying aequorin [49] and carbenicillin resistance genes, using a heat shock method described in [50]. The transformants were selected on Luria bertani (LB) agar containing carbenicillin (300 µg/ml) and verified by PCR using aequorin specific primers (For: 5′CTTACATCAGACTTCGACAACCCAAG, Rev: 5′CGTAGAGCTTCTTAGGGCACAG). Aequorin was expressed and reconstituted as described in [19]. Briefly, mid-log phase cells were induced with IPTG (1 mM) for 2 h for apoaequorin production, and then harvested by centrifugation at 6000 g for 5 min at 4°C. Aequorin was reconstituted by incubating the cells in the presence of 2.5 µM coelenterazine for 30 min.

Luminescence measurements and estimation of free [Ca^2+^]_in_ was performed as described in [19] with slight modifications. Briefly, 100 µl of cells with reconstituted aequorin were equilibrated for 10 min in the dark at room temperature. Luminescence was measured using Synergy Mx Multi-Mode Microplate Reader (Biotek). For basal level of [Ca^2+^]_in_, the measurements were recorded for 1 min at 5 sec interval, then the cells were challenged with 1 mM Ca^2+^, mixed for 1 sec, and the luminescence was recorded for 20 min at 5 sec interval. Injection of buffer alone was used as a negative control, and did not cause any significant fluctuations in [Ca^2+^]_in._ [Ca^2+^]_in_ was calculated by using the formula pCa = 0.612(−log_10_k)+3.745, where k is a rate constant for luminescence decay (s^-1^) [51]. The results were normalized against the total amount of available aequorin as described in [19]. The discharge was performed by permeabilizing cells with 2% Nonidet 40 (NP40) in the presence of 12.5 mM CaCl_2._ The luminescence released during the discharge was monitored for 10 min at 5 sec interval. The estimated remaining available aequorin was at least 10% of the total aequorin. The experimental conditions reported here were optimized to prevent any significant cell lysis.

## Results and Discussion

### *P. aeruginosa* PA4107 Contains Two EF-hand Domains that are Predicted to Localize to the Cell Periplasm

**Figure 1:**
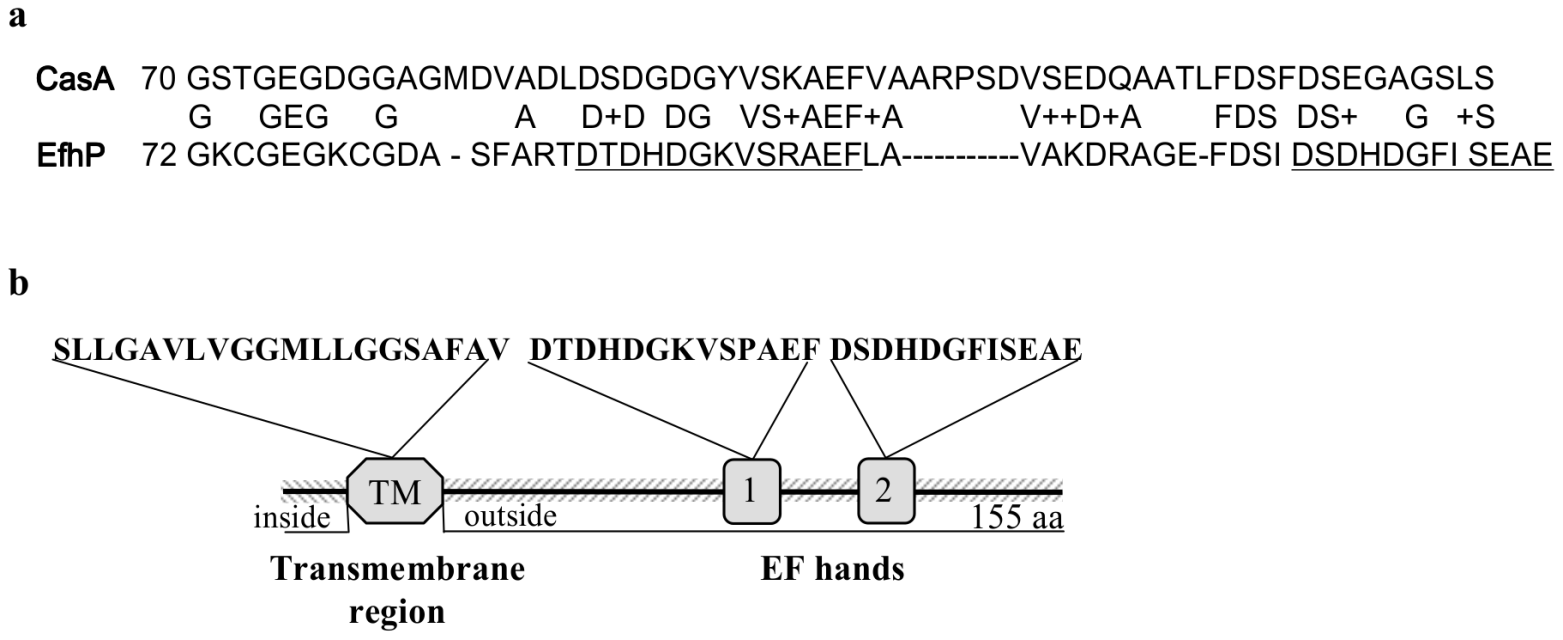
Sequence analyses of EfhP. **a.** Sequence alignment of the EF-hand domains in EfhP (PA4107) and CasA (YP_472788) from *Rhizobium etli* [24]. The predicted Ca^2+^-binding loops are underlined. **b.** The predicted transmembrane (TM) region and two Ca^2+^-binding loops (1 and 2) are shown. EF-hand domains were predicted by PROSITE. Transmembrane region and cellular localization of the protein were predicted by TMHMM.

Based on 17% amino acid sequence identity (over the entire length) with Ca^2+^-binding calsymin (CasA, YP_472788) and the presence of two EF-hand domains with Ca^2+^-binding loops, we predict that the hypothetical protein, PA4107, is a Ca^2+^-binding protein (Fig. 1a) and we designate it EfhP. EfhP contains Ca^2+^-binding loops at positions 88–99, and 115–126. By using PROSITE algorithms, EfhP is also predicted to contain a transmembrane region from amino acids 13–32 (Fig. 1b). Sequence analysis by PrediSI predicted that EfhP is secreted with the signal peptide cleaved at the position 29. However, SignalP 4.0, specifically designed to discriminate signal peptides from transmembrane regions [43], gave low probability that the transmembrane domain is a signal peptide. Analysis by TMHMM indicated that EfhP is an integral membrane protein, with a short N-terminal region located in the cytoplasm, and the majority of the protein, including the C-terminal region and the EF-hands oriented to the periplasm. Based on these analyses, we predict that EfhP spans the inner membrane of *P. aeruginosa* with EF hand domains possibly located in the periplasm.

Sequence analyses of all *P. aeruginosa* proteins indicate that EfhP does not have EF-hand paralogs in the PAO1 genome. Ten completed genomes of *P. aeruginosa, P. putida, P. fluorescens, P. syringae, and P. entomophila* each contain one homolog of EfhP with EF-hand domains. These EF-hand proteins have a wide range of amino acid sequence identity over the length of the proteins, ranging from 19 to 99% (Fig. S1). Multiple sequence alignment revealed 30 highly conserved amino acid residues, 12 of which are within the EF-hand domains. Nine of the homologs are predicted to contain N-terminal transmembrane region. The EF-hand protein of *P. syringae* 1448A has no transmembrane region, and contains four predicted EF-hands domains.

The genome context of *efhP* in *P. aeruginosa* PAO1, suggests that it is operonic with two genes encoding hypothetical proteins PA4106 and PA4105. These proteins contain the DUF692 and DUF2063 domains, respectively, with PA4106 having structural similarity to sugar isomerases, and PA4105 having structural similarity to a predicted transcriptional regulator from *Neisseria gonorrhoeae* (PDB ID: 3DEE). We grouped the genomic environments of *efhP* homologs into 4 groups (Fig. S2). All three *P. aeruginosa* and *P. putida* W619 genomes contain *efhP* homologs adjacent to the DUF692 and DUF2063 domain proteins (Group I). The other three *P. putida* strains and *P. entomophila* L48 genome form group II, where *efhP* homologs are adjacent to genes for the DUF692 and DUF2063 proteins, but with a gene for a hypothetical protein with a DUF4174 domain. The genome context of the *P. fluorescens* Pf0-1 EfhP homologs (Group III) differs from the other Pseudomonads, in that it does not contain downstream genes for DUF692 or DUF2093 protein. Rather, the *P. fluorescens* EfhP homolog is adjacent to upstream genes coding for hypothetical proteins with DUF1780 and DUF3094 domains. The EfhP homolog in *P. syringae* 1448A, in addition to being the most divergent in amino acid sequence, also has a very different genome context, with upstream genes for a histidine kinase and response regulator. Overall, these results indicate that proteins with EF-hand domains are found in all sequenced pseudomonads, but that they have diverged extensively, and therefore may be involved in different Ca^2+^-dependent physiological processes.

### EfhP Contributes to Maintenance of Intracellular Ca^2+^ ([Ca^2+^]_in_) Homeostasis in *P. aeruginosa* PAO1

**Figure 2:**
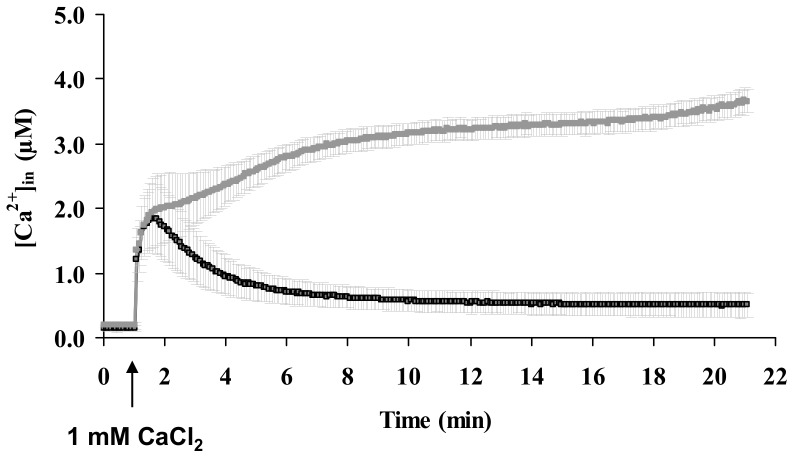
Free [Ca^2+^]_in_ profiles of PAO1 WT (black line) and *efhP* mutant strain PAO1043 (grey line). Cultures were grown in 0^2+^. After the basal level of [Ca^2+^]_in_ was monitored for 1 min, 1 mM Ca^2+^ was added (indicated by the arrow) followed by further [Ca^2+^]_in_ measurement for 20 min.

The role of EfhP in maintaining intracellular Ca^2+^ homeostasis was studied using recombinant Ca^2+^-binding luminescence protein aequorin (**Fig. 2**). The lack of *efhP* did not affect the basal level of [Ca^2+^]_in_ (0.19±0.01 µM) or the initial increase in response to 1 mM Ca^2+^ (1.99±0.14 µM). In contrast, however, this increase was not followed by the recovery of [Ca^2+^]_in_ to nearly basal WT level, as observed in WT PAO1 cells. Instead, the [Ca^2+^]_in_ continued to raise and in 20 min reaching 3.66±0.41 µM, which is almost seven fold higher than in PAO1. This suggests that EfhP is involved in maintaining intracellular Ca^2+^ homeostasis, which supports its predicted Ca^2+^-binding capabilities.

### EfhP Influences the Proteomes of *P. aeruginosa* Cultured at High [Ca^2+^]

**Figure 3:**
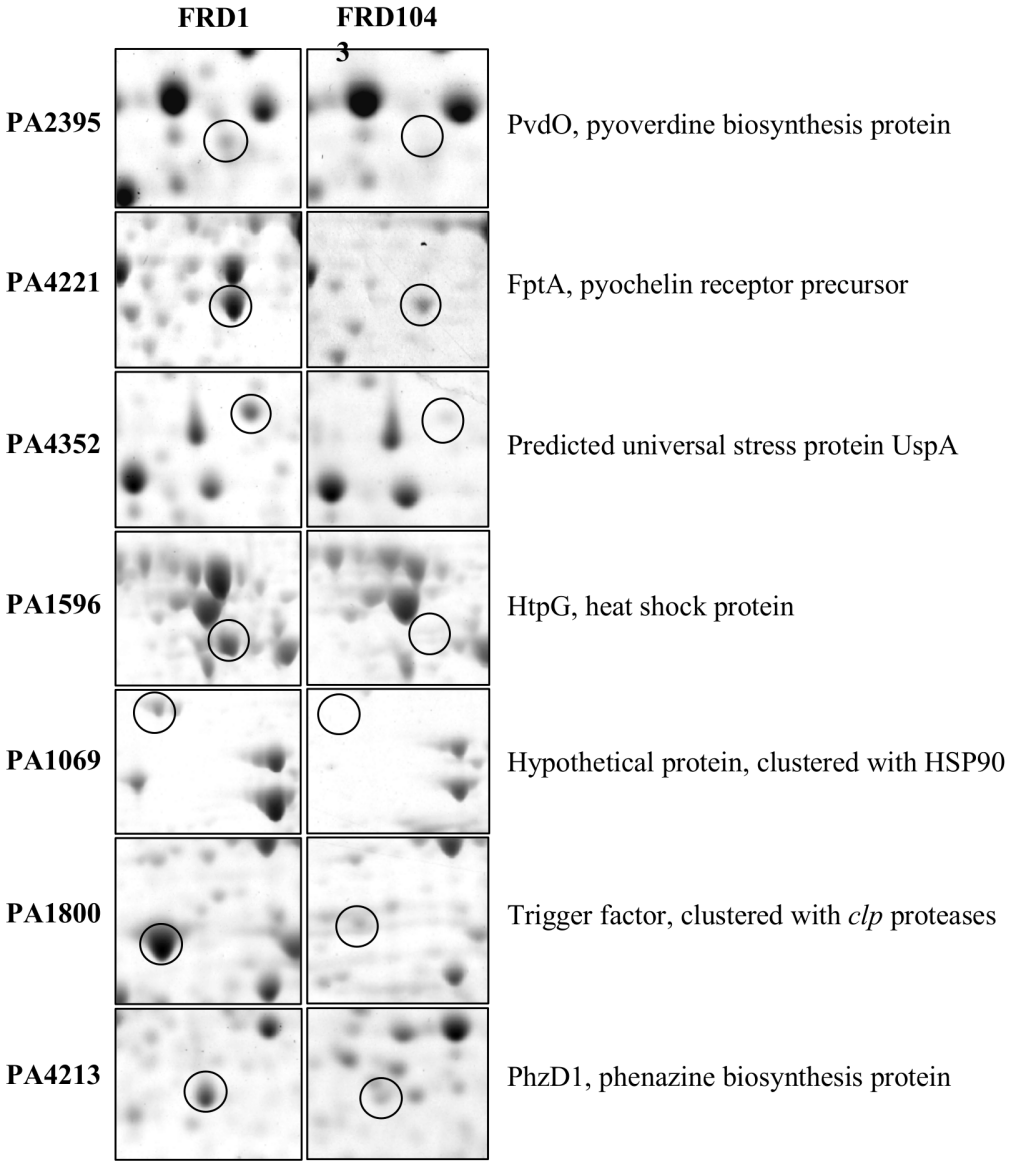
Proteomics analysis of *efhP* mutant strains. Sections of 2D gels showing selected proteins that are affected by the *efhP* mutation in FRD1, cultured in tubing biofilms. Circled are protein missing or with reduced abundances in the *efhP* mutant. Proteins were identified by MALDI-TOF analysis.

In order to determine the role of EfhP in the physiology of *P. aeruginosa*, we performed global quantitative proteomics on wild-type *P. aeruginosa* strains PAO1 and the CF isolate FRD1. We compared those proteomes to the proteomes of their respective *efhP* deletion mutants, designated here as PAO1043 and FRD1043, respectively. Since our previous proteomics studies showed an effect of both [Ca^2+^] and mode of growth (biofilm versus planktonic) on proteome profiles of the wild-type strains [29], we cultured the strains at low [Ca^2+^] (no added Ca^2+^) and high [Ca^2+^] (10 mM), planktonically and in silicon tubing biofilms [52]. Table 1 shows the proteins that were affected by the mutations in at least one strain when the cells were cultured at 10 mM Ca^2+^. Table 1 also indicates whether the protein is found in increased abundance in the wild-type strains during biofilm growth and/or with added Ca^2+^, based on our prior study [29]. Selected proteins are shown in Figure 3.

**Table 1:** Proteins identified by MASCOT-based analysis of MALDI-TOF generated mass spectra.

Protein name	Genename	PAno.	Genetic context	aInduced in biofilm, or byCa^2+^ in PAO1 or FRD1	Normalized signal intensity (NV)^b^ at 10 mM Ca^2+^							
					PAO1		PAO1043		FRD1		FRD1043	
					P^c^	B^d^	P	B	P	B	P	B
**Iron acquiring and storage**												
Pyoverdine biosynthesis protein PvdN	*pvdN*	2394	*pvd* operon	BiofilmCa^2+^	N^e^	0.22	N	**N**	0.05	0.02	**N**	**N**
Pyoverdine biosynthesis protein PvdO	*pvdO*	2395	*pvd* operon	BiofilCa^2+^	N	0.13	N	**N**	0.15	0.09	**N**	**N**
Fe(III)-pyochelin receptor precursor FptA	*fptA*	4221	*fpt*AB and *pchEFR* operons	BiofilmCa^2+^	N	0.19	N	**N**	0.17	0.09	**N**	**0.03**
Ferric iron-binding periplasmic protein HitA	*hitA*	4687	*hit*B	BiofilmCa^2+^	0.16	0.19	0.19	0.15	0.19	0.12	**N**	**N**
Probable binding protein component of ABC Fe transporter		5217	ABC iron transport	Ca^2+^	0.11	0.08	0.09	0.07	0.05	0.02	**N**	**N**
**Stress response**												
Heat shock protein HtpG	*htpG*	1596	Hypothetical	BiofilmCa^2+^	0.51	0.24	**0.24**	0.16	0.38	0.31	0.24	**N**
^f^Predicted universal stress protein UspA		4352	Hypothetical	Biofilm	0.05	0.18	0.12	0.15	N	0.20	0.03	**0.04**
Hypothetical		1069	Heat shock Hsp90	^g^Biofilm Ca^2+^	0.05	0.01	**N**	**N**	0.03	0.13	**N**	**N**
Phosphoenolpyruvate carboxykinase	*pckA*	5192	Heat shock Hsp33	^g^Ca^2+^	0.28	0.2	0.24	**0.04**	0.39	0.3	**0.15**	**N**
**Protein degradation**												
Protease PfpI	*pfpI*	0355	Hypothetical	BiofilmCa^2+^	0.09	0.14	**N**	**0.04**	0.20	0.06	**N**	**N**
Protease IV	*Piv*	4171			N	N	N	N	0.12	0.09	**N**	**N**
Trigger factor	*Tig*	1800	*clp*PX proteases	BiofilmCa^2+^	0.43	0.34	0.41	**0.08**	0.19	0.59	0.19	**0.11**
**Phenazine biosynthesis**												
Phenazine biosynthesis protein PhzB1	*phzB1*	4211	*phz* operon	BiofilmCa^2+^	N	0.09	N	**N**	N	0.19	N	**N**
Phenazine biosynthesis protein PhzD1	*phzD1*	4213	*phz,* operon	BiofilmCa^2+^	0.02	0.20	**N**	**0.03**	N	0.28	N	**0.05**
Phenazine biosynthesis protein PhzB2	*phzB2*	1900	*phz,* operon	BiofilmCa^2+^	N	0.61	N	**N**	0.22	1.15	**N**	**N**

a Based on the data in [69]. ^b^Average normalized volumes over at least two biological replicates under each of the tested growth conditions : ^c^P – planktonic, ^d^B – biofilm. ^e^N - not detected. For protein identification, the MASCOT scores were greater than 75 (p<0.05), and the minimum number of peptides matched was 5. ^f^Protein name was changed based on sequence analysis. ^g^Based on unpublished data. Significant changes in the mutants are shown in bold.

Neither *efhP* mutant strain showed any significant difference in proteome profiles compared to the respective wild-type strain, when cultivated in medium with no added Ca^2+^. However, when cultured with 10 mM Ca^2+^, eight proteins that were abundant in PAO1 were absent in the proteomes of PAO1043 biofilm and/or planktonic cultures: PvdNO, FptA, PA1069, Pfp1, and PhzB1B2D1 (Table 1). The FRD1043 proteome profiles had additional differences compared to the FRD1 profiles when cells were cultured at high [Ca^2+^]. In planktonic cultures, differences included four proteins (HitA, PA1127, PA5217, and Piv) that were absent from the *efhP* mutant. In tubing biofilms, twenty-nine protein differences were observed in the FRD1043 mutant *versus* wild-type strains (Table 1).

### The *efhP* Mutation Influences the Abundance of Proteins Involved in Iron Acquisition

**Figure 4:**
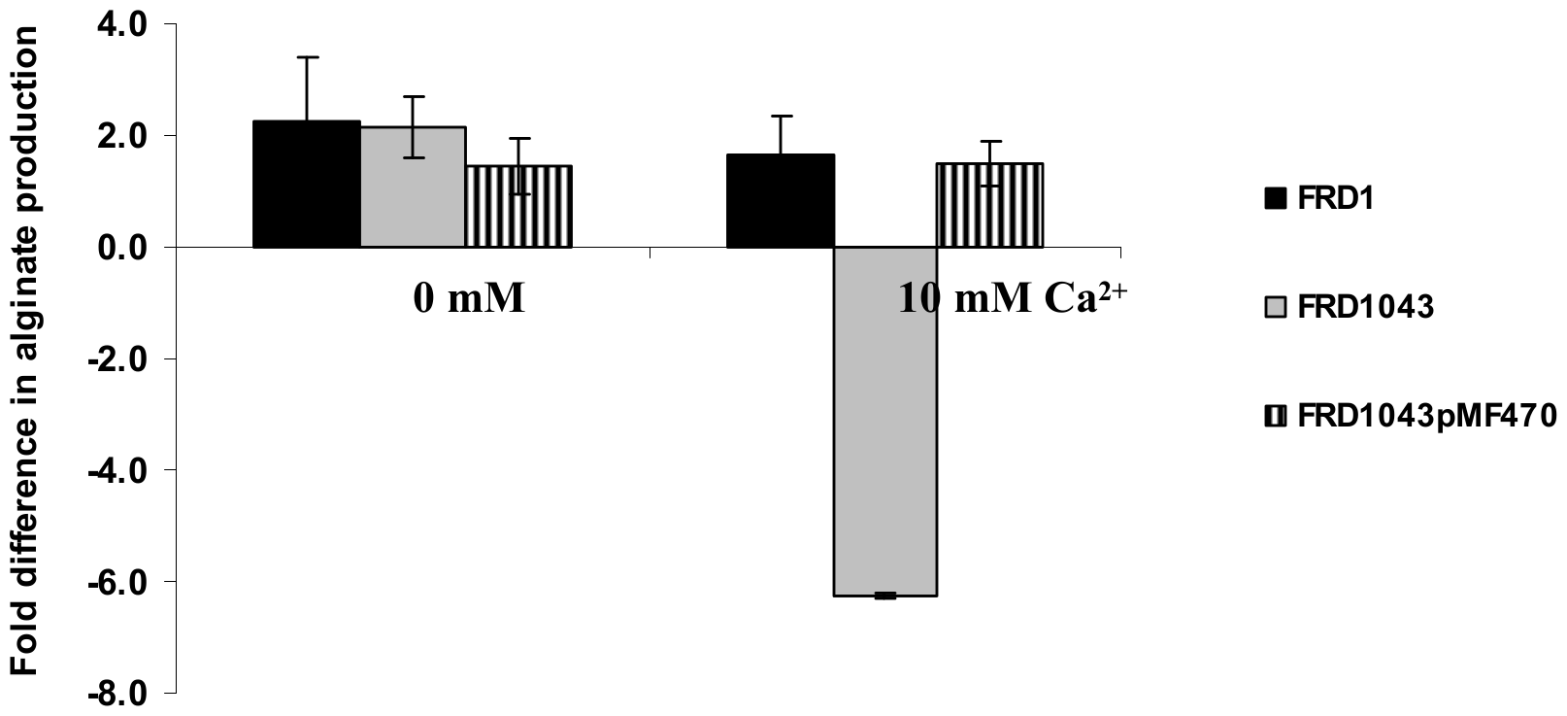
Alginate production at no iron in FRD1 and FRD1043. To deprive the cells of iron, seven passages on no-iron BMM (BMM-NI) agar were performed. The cells were collected using saline. The concentration of alginate was determined using sodium alginate (Spectrum) as a standard and normalized by total cellular protein. Fold difference was calculated between the alginate produced after seventh and first passages. The measurements were obtained for at least three biological replicates, and the mean values with standard deviation are presented.

The role of iron in *P. aeruginosa* pathogenicity is well characterized [53]. Iron limitation in the host is an important signal for inducing biofilm formation [54] and enhancing the expression of virulence factors [55]. Here, we identified five proteins involved in iron acquisition and storage that were less abundant in the mutants cultured at high Ca^2+^ (Table 1, Fig. 3). These include three proteins involved in the two iron-acquisition systems, PvdNO (the high-affinity pyoverdine system) and FptA (the low-affinity pyochelin receptor protein) [56] as well as two iron binding proteins HitA and PA5217. PvdNO and FptA were not expressed in planktonic PAO1, and were essentially absent in both PA1043 and FRD1043 mutants. HitB and PA5217 were present in both wild type strains (biofilm and planktonic), but were not detected in FRD1043 (biofilm and planktonic). Earlier we demonstrated that all of these proteins were highly induced by Ca^2+^ in PAO1 or FRD1 strains [29]. Although the lack of iron did not affect growth of FRD1043 (data not shown), the mutant’s ability to produce alginate was abolished during its biofilm growth when deprived of iron at high Ca^2+^ (Fig. 4). The *efhP* complementation *in trans* fully restored the wild type phenotype. This observation interlinks the regulatory circuits of alginate production with responses to iron starvation and Ca^2+^, and suggests the mediating role of EfhP. We also showed that iron limitation enhances alginate production about twofold in FRD1, which agrees with earlier observations in PAO1 [57] [58] [59].

### The *efhP* Mutations Influence Stress Response Proteins as Well as Oxidative Stress at High [Ca^2+^]

**Figure 5:**
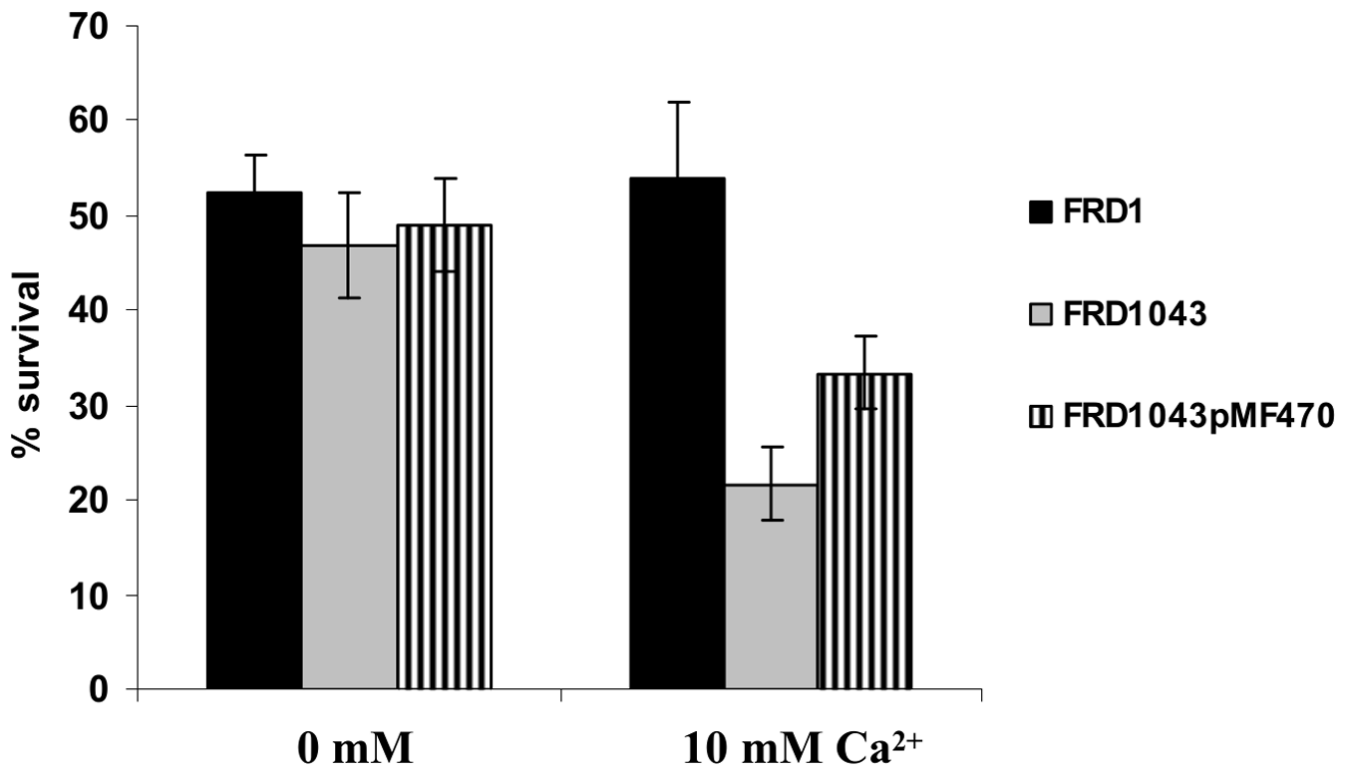
Survival of FRD1 and FRD1043 under oxidative stress. Cells were exposed to 1_2_O_2_ for 1 h at 37°C. Viable cells were determined as colony forming units. Percent survival was calculated considering that non-treated cells are 100% viable. Data represent the mean and standard deviation of at least three biological replicates.

We identified a number of stress response related proteins that were less abundant in the *efhP* mutants than in the wild-type strains (Table 1, Fig. 3). The abundances of these proteins were more impaired in the CF strain FRD1043 than in PAO1043, and more changes occurred in biofilm cultures than in planktonic cells. Stress response proteins that were less abundant in FRD1043 than in FRD1, included the heat shock protein HtpG (PA4352), and the universal stress protein UspA (PA4352). In addition, two proteins PckA and a hypothetical protein, PA1069, were less abundant or not detected in biofilms of both mutants. The genes for the latter two proteins appear operonic with the genes encoding heat shock proteins Hsp33 and Hsp90, respectively. Previously, we showed that all of these proteins were induced by elevated [Ca^2+^] and/or biofilm growth in *P. aeruginosa* [29]. It is possible that Ca^2+^ is recognized as a signal of environmental stress or stress associated with a host environment, and EfhP plays role in the response. To test this hypothesis, we investigated the effect of chemical, oxidative, and heat induced stresses on the mutant strain, FRD1043 in comparison to wild-type, FRD1. Exposing cells to DMSO, ethanol, and heat did not reveal any differences in survival between mutant and wild type strains at either [Ca^2+^] (data not shown). However, the lack of *efhP* impaired the resistance of FRD1 to H_2_O_2_ treatment when cells were cultured in the presence of 10 mM [Ca^2+^], and FRD1043 showed a 60% decrease in survival when exposed to 1 mM of H_2_O_2_ for 60 min (Fig. 5). No significant effect on survival to H2O2 was observed in the *efhP* mutant cultured with no added Ca^2+^. In contrast, wild-type FRD1 cells were equally sensitive to H_2_O_2_ with no added Ca^2+^ and with 10 mM Ca^2+^. Resistance to H_2_O_2_ was partially restored in the *efhP* mutant containing the complementing *efhP* gene *in trans*. The results suggest the importance of EfhP in the ability of *P. aeruginosa* to withstand oxidative stress at elevated Ca^2+^. This resistance to H_2_O_2_-induced oxidative stress may be crucial for survival of *P. aeruginosa* in airways, where H_2_O_2_ is produced in response to infection [60].

### EfhP Affects Production of Virulence Factors, Biofilm Formation, and Virulence in a Plant Infection Model at High [Ca^2+^]

**Figure 6:**
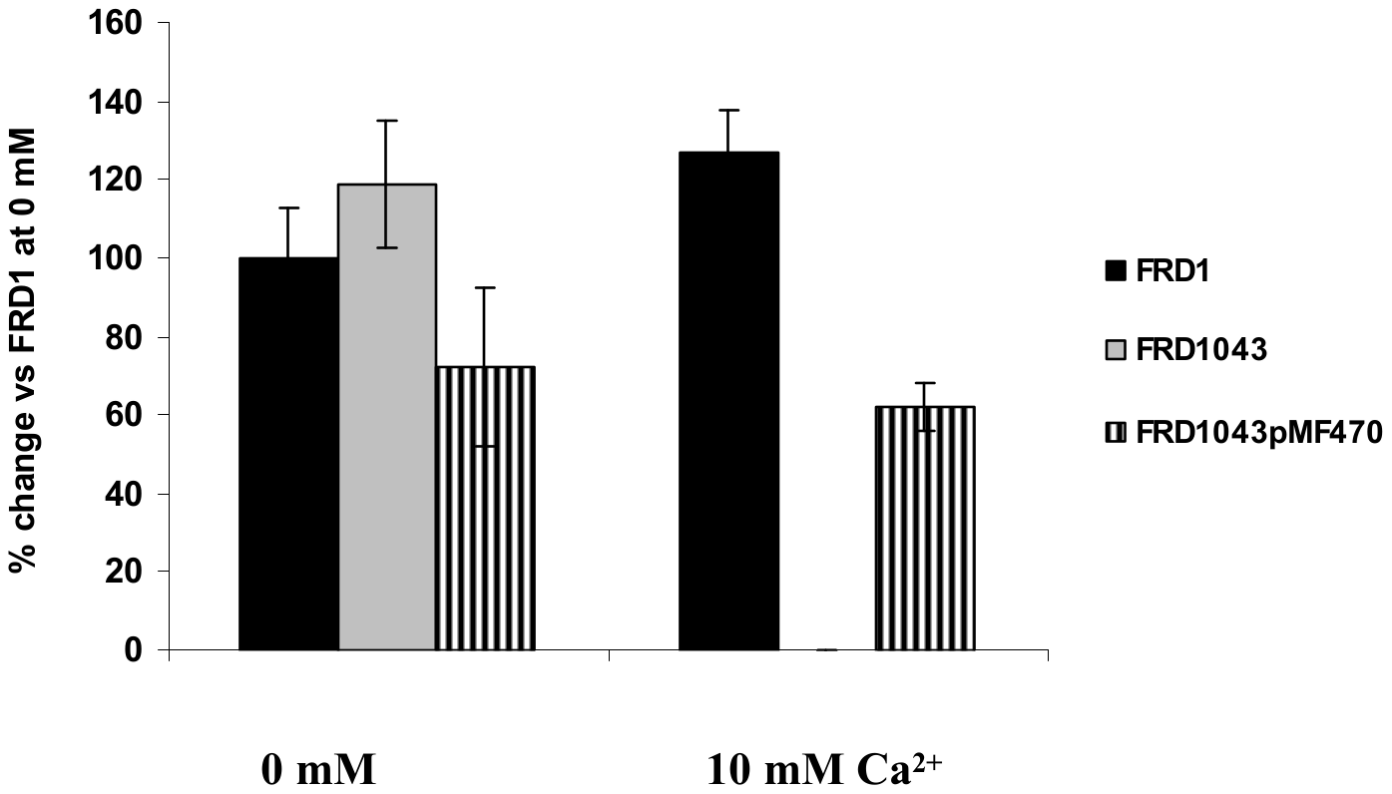
Pyocyanin production of FRD1 and FRD1043. Cells were grown on BMM agar plates at 10_2_ for 24 h, and collected using saline. Percent change was calculated *vs*. FRD1 cells grown at no added CaCl_2_. Data represent the mean and standard deviation of at least three biological replicates.

Pyocyanin is a redox-active virulence factor produced by *P. aeruginosa*. It imposes oxidative stress on airway epithelial cells and mediates tissue damage and necrosis during lung infection [61], [62] [63]. Phenazine biosynthesis proteins PhzB1B2D were at least sixfold less abundant or not detected in the proteomes of strains PA1043 and FRD1043 as compared to PAO1 and FRD1 (Table 1, Fig. 3). These proteins were detected primarily in biofilms of the wild-type strains cultured at high [Ca^2+^] [30] [29], and the mutation-induced effects detected here occurred primarily in biofilm cultures. In agreement, pyocyanin production was not affected in the mutant planktonic cultures (data not shown), but was completely abolished in FRD1043 biofilm cells grown at high [Ca^2+^] (Fig. 6). Biosynthesis of pyocyanin is controlled by the RhlI/RhlR [64] as well as PQS quorum sensing systems [65]. Furthermore, pyocyanin regulation is also affected by iron depletion [66], therefore the effect of EfhP seen here may be related to its effect on iron acquisition enzymes (Table 1) or its potential interaction with one of several major regulatory systems in *P. aeruginosa* (reviewed in [67]).

We previously showed that several proteases are induced by high [Ca^2+^], and that extracellular proteases accumulate in the matrix material of FRD1 biofilms [30]. Two proteases Piv, PfpI were at least fourfold less abundant in PA1043 than in PAO1, and were not detected in FRD1043 planktonic and biofilm cells (Table 1). In addition, a trigger factor (PA1800), encoded on the same operon as the *clpP*, *clpX*, and *lon* proteases had reduced abundance in the PA1043 and FRD1043 mutant strains (Fig. 3). Since secreted proteases are harbored in the biofilm matrix of *P. aeruginosa* [30], the decreased abundance of proteases in the mutant strain biofilms may be related to the mutant’s reduced ability to grow a biofilm. Earlier we have shown that Ca^2+^ enhances biofilm formation in *P. aeruginosa* [30], [29]. To test whether EfhP plays a role in this induction, FRD1, its *efhP* mutant, and complemented strain were grown in biofilms using 96-well plates. The lack of *efhP* caused 35% decrease in the organism’s ability to form biofilm, however complementation of the gene did not restore the wild type phenotype (Fig. S3). The latter may be due to the different level of *efhP* expression in the complemented strain compared to the wild type, which may be particularly influential since the protein plays role in maintaining intracellular Ca^2+^ homeostasis. We also cannot rule out that the mutant strain acquired a compensatory mutation that cannot be complemented. Finally, the reducing effect of the *efhP* deletion on biofilm growth coincided with a similar effect on H_2_O_2_ resistance and pyocyanin production. Both of the latter phenotypes are induced during biofilm growth [29] [68] and were restored by the *efhP* complementation.

**Figure 7:**
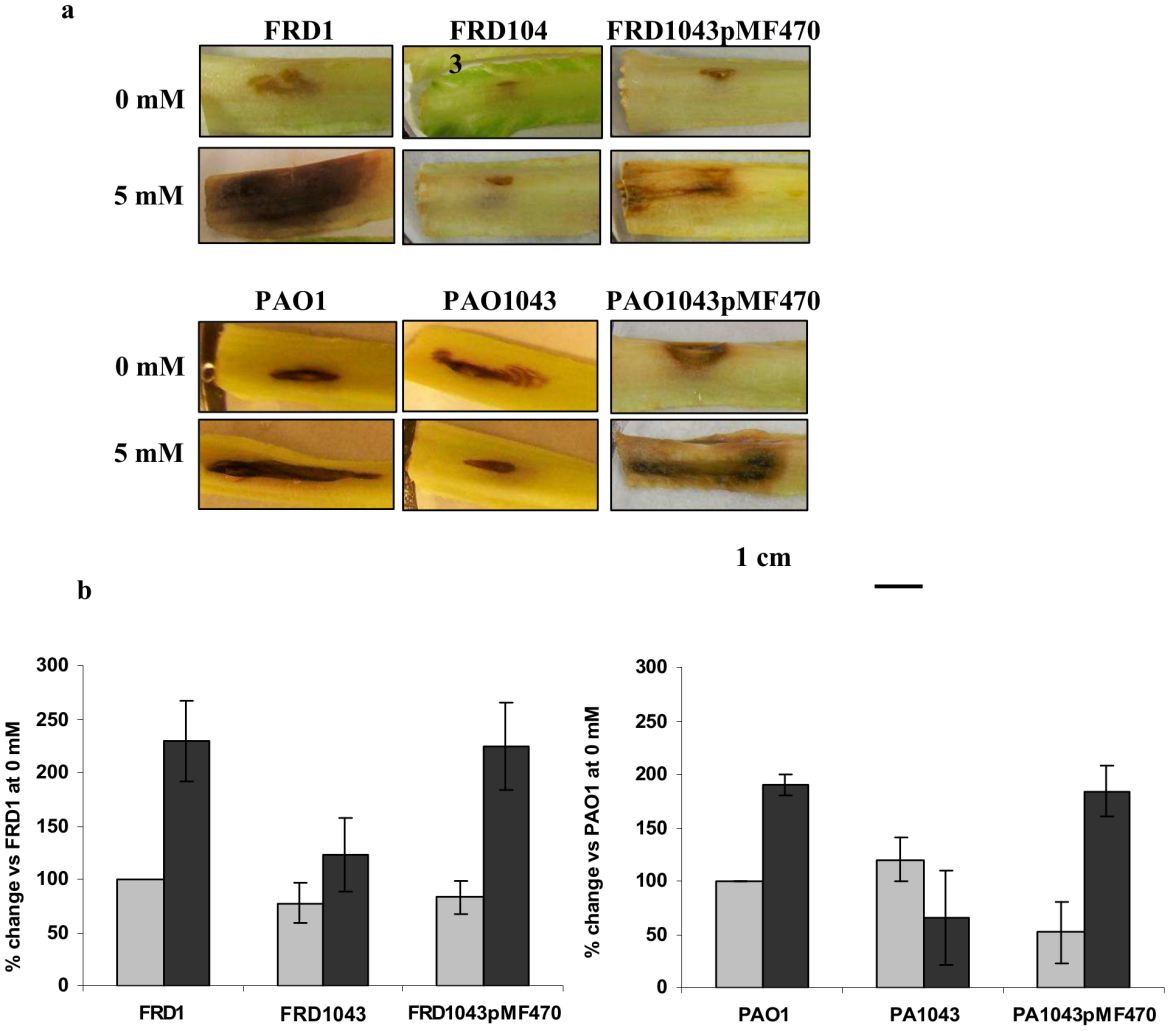
Lettuce infection assay of *P. aeruginosa* virulence. **a**. Photographs showing representative samples of lettuce midribs after 6 days of infection with FRD1 and PAO1 and their *efhP* mutant strains FRD1043 and PAO1043. Complementing with *efhP* in trans on plasmid pMF470 partially restored the zone of infection. **b**. The areas of disease were measured and the percent change *vs*. wild types was calculated. The experiments were repeated at least two times, with 3–5 biological replicates each.

To determine if EfhP plays a role in *P. aeruginosa* virulence when the bacteria are exposed to high [Ca^2+^], we used the lettuce infection model [48] and compared infection zones in the wild-type and the *efhP* mutant strains. In both PAO1 and FRD1, preincubation with 5 mM CaCl_2_ resulted in increased zone of disease, compared to bacteria cultured with no added CaCl_2_ (Fig. 7). This effect of Ca^2+^ was more pronounced in FRD than in PAO1. When cultured at low [Ca^2+^], little effect of the *efhP* mutation was observed for both strains. However, when the strains were cultured with 5 mM CaCl_2_, the zone of disease reduced at least two fold in the mutants compared to the wild-type strains. The disease zone was partially restored in the mutant cells complemented with *efhP* in *trans*. These results suggest that EfhP is required for Ca^2+^-induced virulence of *P. aeruginosa* in a plant model, likely due to the effect of EfhP on virulence factor production at high [Ca^2+^].

The *P. aeruginosa* EfhP has sequence similarity to the EF-hand protein, CasA, produced by the plant symbiont, *Rhizobium etli*. *R. etli casA* is expressed during plant host invasion and is required for symbiotic nitrogen fixation [24]. Therefore we hypothesize that EfhP of *P. aeruginosa* may also play a role in Ca^2+^-dependent interaction between *P. aeruginosa* and its host during infection. Based on the microarray expression data available from the GEO database, the transcription of *efhP* increases more than 150 fold in *P. aeruginosa* isolates from CF sputa grown both *in vitro* and *in vivo* (GDS2869 and GDS2870). Considering that Ca^2+^ accumulates in pulmonary liquids of CF patients [10], this provides further evidence for a role of EfhP in *P. aeruginosa* adaptation to a host environments with elevated [Ca^2+^].

### Conclusions

Sequence analyses predicted that EfhP is a Ca^2+^-binding protein spanning the inner membrane, with the two EF-hand domains facing the periplasm. Deletions of e*fhP* in two *P. aeruginosa* strains causes multiple changes in the cytosolic proteome of both strains, but with more changes occurring in the CF pulmonary isolate FRD1. The effects of the *efhP* deletions only occurred when the cells were exposed to elevated [Ca^2+^] and included reduced abundance of virulence factors and stress response proteins. The lack of e*fhP* abolished production of pyocyanin, and reduced the degree of infection, biofilm formation, and resistance to oxidative stress in FRD1 at high [Ca^2+^]. The mutant also lost the ability to produce alginate at no iron and high [Ca^2+^]. Finally, the lack of EfhP abolished the ability of *P. aeruginosa* to maintain intracellular Ca^2+^ homeostasis. These findings suggest that EfhP is important for Ca^2+^ homeostasis and plays role in Ca^2+^- triggered virulence and resistance of *P. aeruginosa* in high Ca^2+^ environments.

## Supporting Information

Figure S1 **Sequence alignment of EfhP and its homologs from nine** ***Pseudomonas*** **strains.** EF-hands are shown in boxes, transmembrane regions are shown in grey. Conserved amino acids are in bold. Sequences were aligned using ClustalW.
(TIF)

Figure S2 **Genome context of the** ***efhP*** **homologs from Pseudomonads.** The organization of the *efhP* genomic neighborhood in *P. putida strains* KT2440, F1, W619, and GB-1, *P. entomophila* L48, *P. aeruginosa* strains LESB58, PAO1, and PA7, *P. fluoresecens* Pf0-1, and *P. syringae* 1448A. Genes are colored according to their predicted functional domains: black, EfhP and homologs; wavy lines, DUF domain proteins; horizontal lines, transcriptional regulators; diagonals, two component system; light grey, hypothetical proteins.
(TIF)

Figure S3 **Biofilm assessment.** Biofilm formation was quantified using 96-well plates. Cells were cultured on BMM medium containing either 10 mM CaCl2 or no added calcium for 24 h with medium replacement at 12 h. Biofilms were stained with crystal violet, and the absorbance was measured at 595 nm using a microtiter plate reader.
(TIF)
